# Prevalence and phenotype associations of complement factor I mutations in geographic atrophy

**DOI:** 10.1002/humu.24242

**Published:** 2021-06-29

**Authors:** Adnan H. Khan, Janice Sutton, Angela J. Cree, Samir Khandhadia, Gabriella De Salvo, John Tobin, Priya Prakash, Rashi Arora, Winfried Amoaku, Peter Charbel Issa, Robert E. MacLaren, Paul N. Bishop, Tunde Peto, Quresh Mohamed, David H. Steel, Sobha Sivaprasad, Clare Bailey, Geeta Menon, David Kavanagh, Andrew J. Lotery

**Affiliations:** ^1^ Division of Clinical Neurosciences, Clinical and Experimental Sciences, Faculty of Medicine University of Southampton Southampton UK; ^2^ Southampton Eye Unit University Hospital Southampton NHS Foundation Trust Southampton UK; ^3^ Gyroscope Therapeutics Limited Stevenage UK; ^4^ The Eye Unit The Princess Alexandra Hospital NHS Trust Harlow UK; ^5^ Department of Ophthalmology Salisbury District Hospital, Salisbury NHS Foundation Trust Salisbury UK; ^6^ Eye and ENT Centre, Queen's Medical Centre Nottingham University Hospitals NHS Trust Nottingham UK; ^7^ Oxford Eye Hospital and Oxford NIHR Biomedical Research Centre, John Radcliffe Hospital Oxford University Hospitals NHS Foundation Trust Oxford UK; ^8^ Nuffield Laboratory of Ophthalmology, Nuffield Department of Clinical Neurosciences University of Oxford Oxford UK; ^9^ Division of Evolution and Genomic Sciences, Faculty of Medicine and Health, School of Biological Sciences University of Manchester Manchester UK; ^10^ Manchester Royal Eye Hospital, Manchester University NHS Foundation Trust Manchester Academic Health Science Centre Manchester UK; ^11^ Centre for Public Health, School of Medicine, Institute of Clinical Sciences Queen's University Belfast Belfast UK; ^12^ Department of Ophthalmology, Gloucestershire Royal Hospital Gloucestershire Hospitals NHS Foundation Trust Gloucester UK; ^13^ Sunderland Eye Infirmary South Tyneside and Sunderland NHS Foundation Trust Sunderland UK; ^14^ Biosciences Institute Newcastle University Newcastle upon Tyne UK; ^15^ Institute of Ophthalmology University College London London UK; ^16^ Clinical Research Unit, Bristol Eye Hospital University Hospitals Bristol NHS Foundation Trust Bristol UK; ^17^ Department of Ophthalmology, Frimley Park Hospital Frimley Health NHS Foundation Trust Camberley UK; ^18^ National Renal Complement Therapeutics Centre Royal Victoria Infirmary Newcastle upon Tyne UK; ^19^ Translational and Clinical Research Institute Newcastle University Newcastle upon Tyne UK

**Keywords:** age‐related macular degeneration, complement factor I, factor I, geographic atrophy, reticular pseudodrusen

## Abstract

Rare variants in the complement factor I (*CFI*) gene, associated with low serum factor I (FI) levels, are strong risk factors for developing the advanced stages of age‐related macular degeneration (AMD). No studies have been undertaken on the prevalence of disease‐causing *CFI* mutations in patients with geographic atrophy (GA) secondary to AMD. A multicenter, cross‐sectional, noninterventional study was undertaken to identify the prevalence of pathogenic rare *CFI* gene variants in an unselected cohort of patients with GA and low FI levels. A genotype‐phenotype study was performed. Four hundred and sixty‐eight patients with GA secondary to AMD were recruited to the study, and 19.4% (*n* = 91) demonstrated a low serum FI concentration (below 15.6 μg/ml). *CFI* gene sequencing on these patients resulted in the detection of rare *CFI* variants in 4.7% (*n* = 22) of recruited patients. The prevalence of *CFI* variants in patients with low serum FI levels and GA was 25%. Of the total patients recruited, 3.2% (*n* = 15) expressed a *CFI* variant classified as pathogenic or likely pathogenic. The presence of reticular pseudodrusen was detected in all patients with pathogenic *CFI* gene variants. Patients with pathogenic *CFI* gene variants and low serum FI levels might be suitable for FI supplementation in therapeutic trials.

## Graphical abstract

This study demonstrates, for the first time, the prevalence of disease‐causing complement factor I (CFI) gene mutations in an unselected cohort of patients with geographic atrophy secondary to age‐related macular degeneration. Reticular pseudodrusen was detected in all patients with a rare *CFI* gene variant in this study.

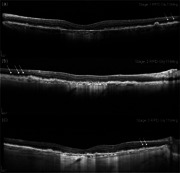

## 1 INTRODUCTION

Age‐related macular degeneration (AMD), a progressive retinal disease that results in the loss of central vision, is predicted to affect 288 million people worldwide by 2040 (Wong et al., 2014). Atrophic (dry) AMD is believed to be caused by progressive degeneration of retinal pigment epithelium (RPE) cells and choroid, leading to secondary photoreceptor damage and eventually, the clinical phenotype of geographic atrophy (GA). Neovascular, or wet AMD, is a result of choroidal neovascularisation, resulting in rapid vision loss (Khandhadia et al., 2012). A method to identify asymptomatic patients who are at the highest risk of developing the sight‐threatening, advanced stages of the disease, has been a common goal amongst the ophthalmology community with the aim of therapeutic intervention at the asymptomatic stage.

AMD has a complex multifactorial etiology, influenced by age, genetics, environment, and possibly diet (Lim et al., 2012). The evidence base for a genetic component is significant, and many single nucleotide polymorphisms (SNPs) have been associated with a patient's risk of developing AMD (Fritsche et al., 2016). AMD‐associated SNPs in genes of alternative complement pathway components, including the complement factor B (*CFB*) gene region (Shuai et al., 2017) and the *C3* gene (Maller et al., 2007), have been reported. Common and rare genetic variants at the regulators of complement activation locus on *chromosome* 1, which contains the *CFH* and *CFHR* genes, contribute to AMD risk (Cipriani et al., 2020; Edwards et al., 2005; Fritsche et al., 2016; Haines et al., 2005), in addition to a number of genetic variants in the complement factor I (*CFI*) gene region on *chromosome* 4 (Alexander et al., 2014; Fagerness et al., 2009). SNPs in genes independent of the complement pathway can also be associated with AMD risk. *ARMS2* is one of the major susceptibility genes for AMD (Rivera et al., 2005), with odds ratios similar to those observed with *CFH* gene variants of the complement pathway.

The complement factor I (factor I [FI]) protein, encoded by the *CFI* gene, is a normal plasma component whose function is to downregulate the alternative complement pathway via cleavage of C3b into inactive iC3b in the presence of its cofactors (Lachmann, 2019). It has been reported that a collection of complement risk SNPs for AMD, or “complotype”, alters the downregulation of the C3b feedback cycle by FI (Lay et al., 2015). In addition to the common risk *CFI* SNPs described above, next‐generation sequencing studies have identified an increasing number of rare *CFI* gene variants that are associated with AMD development (Seddon et al., 2013). Initial efforts to understand the functional effects of *CFI* gene variants focused on circulating levels of the FI protein and demonstrated that rare *CFI* gene variants associated with low levels of FI were strong risk factors for AMD (Kavanagh et al., 2015). This has been replicated in more recent studies with low serum concentrations of FI being detected in patients with the more advanced stages of AMD (de Jong et al., 2020; Hallam et al., 2020). One such example is the p.Gly119Arg (NM_000204.3:c.355G>A) substitution, which confers both a high odds ratio of AMD risk (odds ratio = 22.20) and reduced expression of FI (van de Ven et al., 2013). It has been suggested that FI supplementation might be a logical and practical method to downregulate the alternative complement pathway in AMD, including its associated hyperinflammatory response involving the RPE and local vasculature (Lachmann, 2019). Furthermore, since FI is a normal plasma component, there would be no risk of immunogenicity from increased concentrations (Lachmann, 2019).

The aims of this study were to (1) identify, for the first time, the prevalence of pathogenic *CFI* gene variants in an unselected cohort of GA patients; and (2) report the detailed phenotype of these patients. The results of this study should therefore inform what proportion of GA patients might have FI disease‐causing mutations. This would be useful information for the design of future therapeutic trials.

## 2 METHODS

### 2.1 Study approval, registration, and regulation

This study was conducted in accordance with the Research Governance Framework for Health and Social Care (2005) and Good Clinical Practice. Ethical approval was obtained from Yorkshire & The Humber‐South Yorkshire Research Ethics Committee. This study adhered to the tenets of the Declaration of Helsinki. The University Hospital Southampton NHS Foundation Trust was the sponsor of this study, and The University of Southampton, through its Faculty of Medicine, undertook the research study. All patient samples and data were anonymized for the purpose of this study. Patient DNA and serum samples were stored for future studies. Procedures for handling, processing, and storage of patient data were in compliance with the UK Data Protection Act (1998). This study is registered on a publicly accessible database: The NIHR Clinical Research Network Portfolio ID 34996.

### 2.2 Patient recruitment, consent, inclusion and exclusion criteria

Twelve hospitals in the United Kingdom identified patients with a diagnosis of GA secondary to AMD from routine outpatient clinics, or invited patients from their local databases, to participate in this study. Informed consent was obtained from all patients before inclusion in the study. The main inclusion criterion for this study was the diagnosis of GA secondary to AMD. The exclusion criteria for this study were: (1) any other ocular disease, which could mask the condition being studied (GA secondary to AMD) due to confounding pathology; and (2) any history of neovascular (wet) AMD in either eye.

### 2.3 Patient investigation

All patients recruited to this study with a diagnosis of GA were invited for an initial visit where their demographics and medical history were recorded. Patients provided blood samples for measurement of serum FI and C‐reactive protein (CRP) concentrations, and for potential DNA sequencing. Patient blood samples were collected and shipped in dry ice to Eurofins Central Laboratories. Kits for collection and transport of study samples to the laboratory were provided by Eurofins.

### 2.4 Serum FI detection

Serum FI concentrations were measured commercially by Eurofins Central Laboratory, using enzyme‐linked immunosorbant assay (ELISA) (Hycult Biotech).

The lower limit of normal was defined as the lower 2.5th percentile of serum FI measured from 159 AMD subjects (13.1 μg/ml) plus 18% of this value to allow for assay variation, giving a final threshold of 15.6 μg/ml.

### 2.5 Genetic analysis

If patient serum FI concentrations were detected to be low (below 15.6 μg/ml), DNA sequencing of the *CFI* gene was undertaken to investigate if rare *CFI* gene variants were present. Although there is no consensus in the literature on the minor allele frequency (MAF) of a rare genetic variant, for the purposes of this study, a rare genetic variant was described as having an MAF of less than 1%, as defined previously (Frazer et al., 2009; Saint Pierre & Genin, 2014). *CFI* genetic analysis was undertaken by the Northern Molecular Genetics Service (a UKAS Accredited Testing Laboratory No. 9028), The Newcastle upon Tyne Hospitals NHS Foundation Trust, Newcastle upon Tyne, UK, as previously described (Gleeson et al., 2016; Kavanagh et al., 2005). Sequence analysis was performed using the Mutation Surveyor software (v4.0.8), (SoftGenetics LLC) on all coding exons ±10 bases of the *CFI* gene and the following 10 susceptibility SNPs associated with AMD: *CFH* region: rs800292 (NM_000186.4:c.184G>A); rs1061170 (NM_000186.4:c.1204C>A); rs10737680 (NC_000001.11:g.196710325A>C); rs1329428 (NC_000001.11:g.196733680C>T); *CFI* region: rs17440077 (NC_000004.12:g.109616411A>G); rs4698775 (NC_000004.12:g.109669323G>T); rs2285714 (NM_030821.5:c.345G>A); *CFB* region: rs429608 (NC_000006.12:g.31962685G>A); *C3* region: rs2230199 (NM_000064.4:c.304C>G); *ARMS2* region: rs10490924 (NM_001099667.3:c.205G>T). The expected false‐negative rate was less than 1%. Minor allele frequencies of rare *CFI* gene variants quoted in this study were based on population frequencies (European, non‐Finnish) according to the genome aggregation database (gnomAD): http://gnomad.broadinstitute.org.

### 2.6 Patient imaging and assessment

Patients with both a low serum FI level and a confirmed *CFI* gene variant on DNA sequencing were invited back for a second hospital visit to undergo a dilated retinal examination and retinal imaging. The imaging consisted of spectral domain optical coherence tomography (SD‐OCT), fundus autofluorescence (both recorded with a Spectralis HRA‐OCT, Heidelberg Engineering, Heidelberg, Germany), and color fundus photographs. All patient retinal images were submitted to the University of Southampton, where they were reviewed by two ophthalmologists (AHK and GDS) masked to the results of *CFI* gene sequencing. A third ophthalmologist (AJL), also masked to the results of gene sequencing, reviewed specific patient retinal images in the event of a lack of agreement during the initial imaging review. The presence of reticular pseudodrusen (RPD) on retinal imaging was based on the four stages of RPD described by Querques et al. (2012; 2013).

### 2.7 Statistical analyses

The GraphPad Prism software version 8.2 (GraphPad Software) was used for statistical analyses and graphical representation of the data obtained in this study.

The unpaired *t* test with Welch's correction was used to determine statistically significant differences in age, number of SNPs at 10 common AMD loci, and average serum FI and CRP concentrations between groups. Statistical significance was set at the *p* < .05 value. Single variant association studies with 2 × 2 contingency tables were used to determine statistically significant associations between factors in this study: these include the presence of *CFI* gene variants and association with the sex of the patient, and the presence of RPD associated with *CFI* gene variants. A Bonferroni correction was employed in the event of multiple analyses from the same pool of data. Both the Pearson rank and linear regression tests were employed to determine correlation and regression between variables in this study.

## 3 RESULTS

### 3.1 Serum FI and CRP concentrations in patients with GA and rare *CFI* gene variants

**Figure 1:**
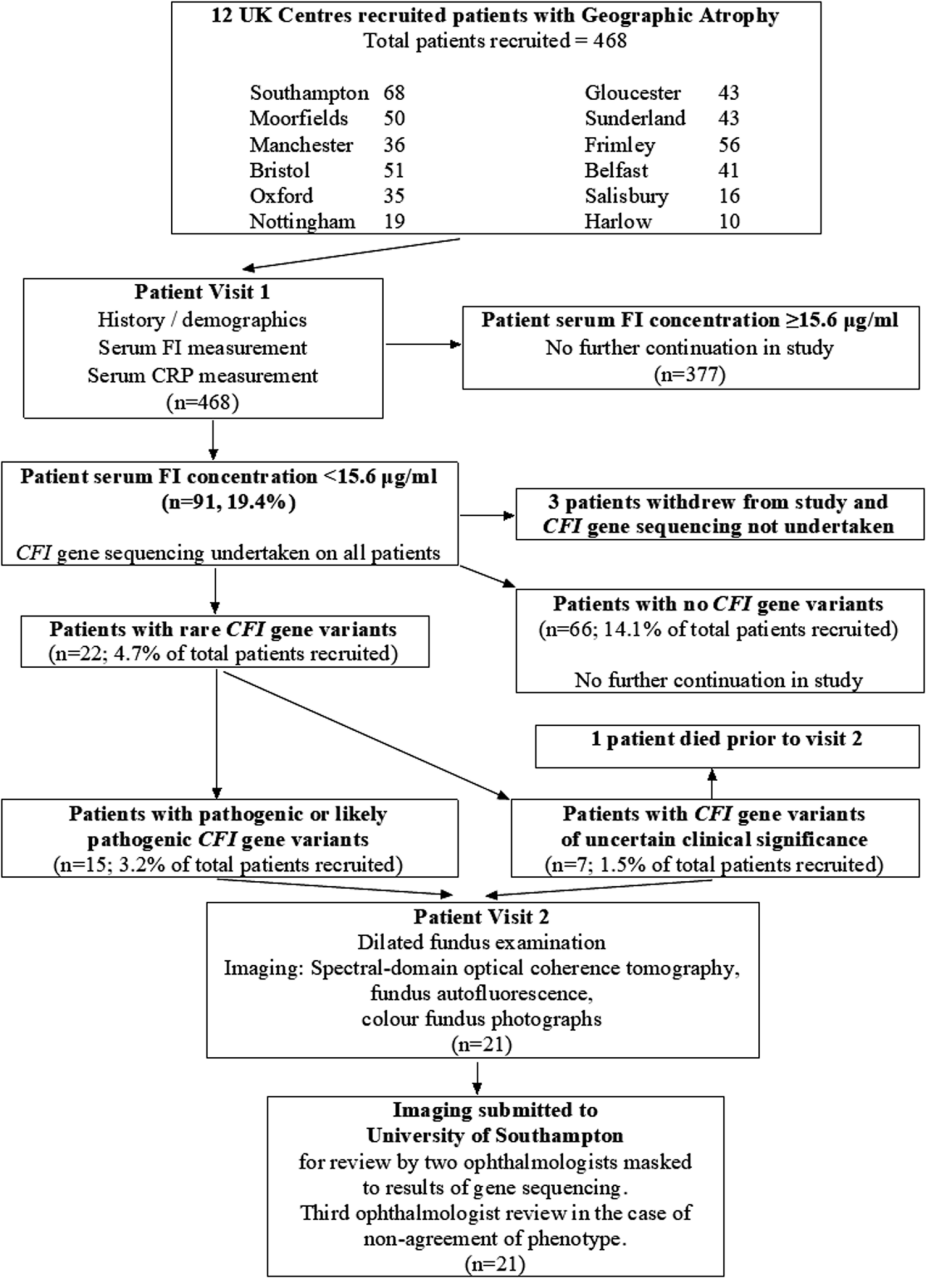
A flowchart diagram summarizing the investigation pathway of 468 patients with geographic atrophy (GA) recruited to this multicenter study. Patients attending for a 2nd visit were those with low serum factor I (FI) concentration, defined in this study as less than 15.6 μg/ml, in whom a *CFI* gene variant was detected after *CFI* gene sequencing. CFI, complement factor I

**Figure 2:**
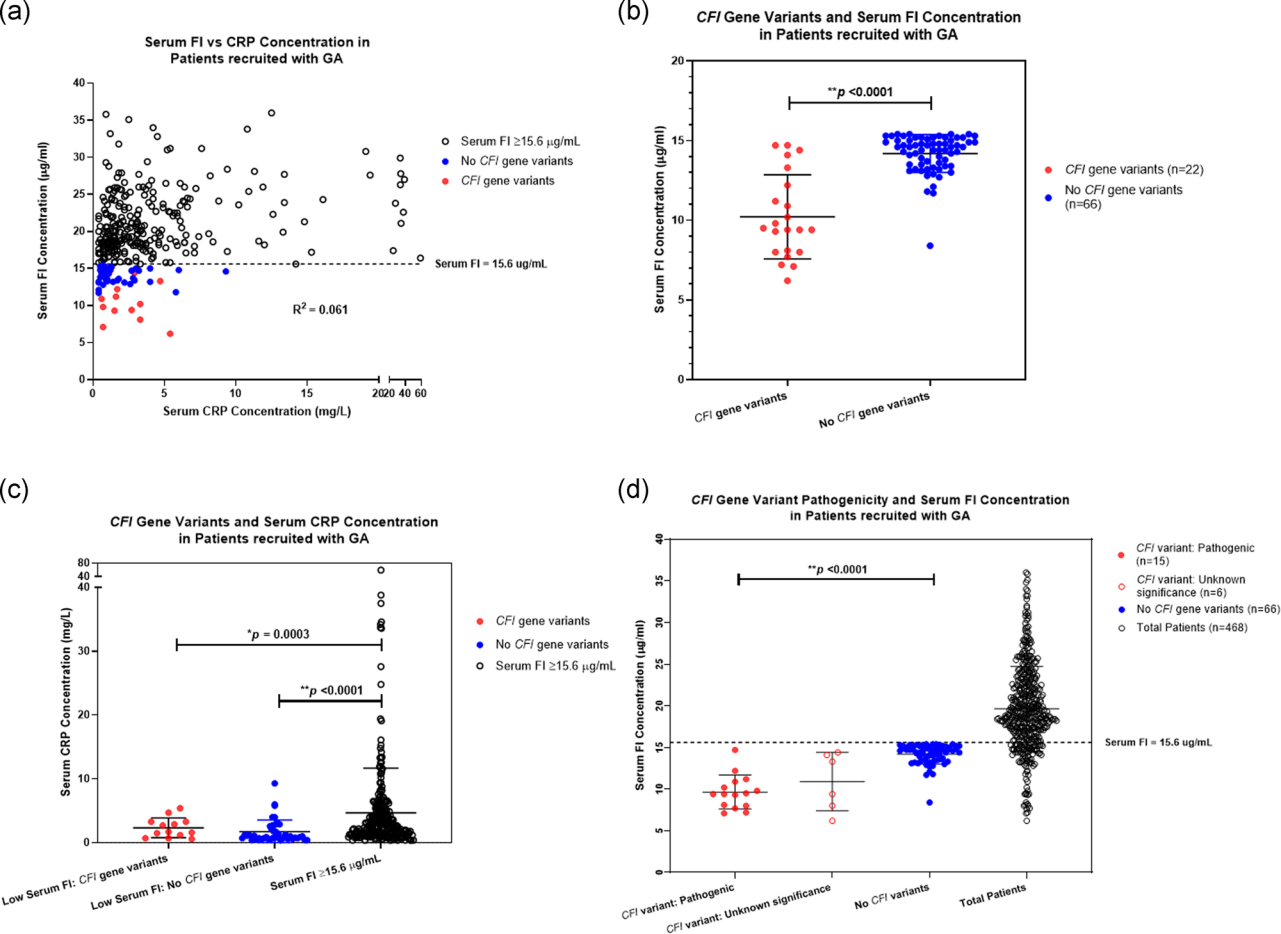
All patients with geographic atrophy recruited to this study (*n* = 468) underwent measurement of serum concentrations of factor I (FI) and C‐reactive protein (CRP). A low FI concentration was defined in this study as less than 15.6 μg/ml, represented on the graph by the horizontal dotted line. The serum concentration values of patients with a clinically detectable level of CRP (CRP: >0.3 mg/L; *n* = 322) are plotted against serum FI concentration values on the graph (a); patients with serum FI concentration greater than or equal to 15.6 μg/ml denoted by a white circle with black outline; patients with a low serum FI concentration and detection of a *CFI* gene variant denoted by a solid red circle; patients with a low serum FI concentration and no detection of a *CFI* gene variant denoted by a solid blue circle. A linear regression test was carried out, with the *R* ^2^ value shown, to determine the relationship of the two variables, in addition to a Pearson correlation test. Patients with low serum FI concentrations underwent *CFI* gene sequencing to detect *CFI* gene variants. A total of 21 patients with low serum FI concentration expressed rare *CFI* gene variants compared to 66 patients who did not. The mean ± *SD*s of patient serum FI concentrations are plotted on a graph (b), according to the status of *CFI* gene variant expression (patients with detection of *CFI* gene variants denoted by a solid red circle; patients with no detection of *CFI* gene variants denoted by solid blue circle). The mean ± *SD*s of serum CRP concentrations of patients are also plotted on a graph (c) based on serum FI concentration (below or ≥15.6 μg/ml), and in the case of low serum FI level, the status of *CFI* gene variant expression (patients with serum FI concentration ≥15.6 μg/ml denoted by a white circle with black outline; patients with a low serum FI concentration and detection of a *CFI* gene variant denoted by a solid red circle; patients with a low serum FI concentration and no detection of a *CFI* gene variant denoted by a solid blue circle). Classification of the likely pathogenicity of the rare *CFI* gene variants detected was based on bioinformatic prediction and American College of Medical Genetics and Genomics (ACMG) guidelines. The mean ± *SD*s of serum FI concentrations of those patients with a pathogenic *CFI* gene variant, a *CFI* gene variant of unknown clinical significance, and patients with no *CFI* gene variants are shown on the graph (d) (patients with a low serum FI level and pathogenic *CFI* gene variant denoted by a solid red circle; patients with a low serum FI level and *CFI* variant of unknown clinical significance denoted by a white circle with red outline; patients with a low serum FI level and no *CFI* gene variants denoted by a solid blue circle; all patients regardless of detection of *CFI* gene variant denoted by a white circle with black outline). The unpaired *t* test, two‐tailed, with Welch's correction, was used to determine whether there was a statistically significant difference in serum FI or CRP concentrations between groups. *p<.05; **p<.0001. CFI, complement factor I; FI, factor I

A total of 468 patients with a diagnosis of GA were recruited from 12 UK hospitals to take part in this study (Figure 1). The mean age of patients recruited to the study was 79.1 [*SD* = 8.1]. Of those recruited, 36.1% (*n* = 169) were male and 63.9% (*n* = 299) were female. Subjects' serum FI concentrations ranged from 6.2 to 36 μg/ml, with a mean concentration of 19.6 μg/ml (*SD* = 5.1; *n* = 468). There was minimal skew to the data. Serum CRP concentrations ranged from 0.3 to 59 mg/L, with a mean concentration of 4.2 (*SD* = 6.5; *n* = 322). The demographics of the patients and key clinical findings of the study are summarized in Table 1. All recruited patients with a clinically detectable serum CRP concentration (≥0.3 mg/L; *n* = 322) and corresponding serum FI concentration were plotted on a graph (Figure 2a). The Pearson rank test was used to investigate a relationship between serum FI and CRP concentrations in patients with GA in this study. This showed a statistically significant positive correlation with Pearson correlation coefficient *r* value = .2464 (*p* < .0001). Linear Regression testing demonstrated weak goodness of fit with *R* ^2^ = .06072.

**Table 1:** Complement factor I in geographic atrophy study: demographics and summary table

Number of patients recruited to study	468
Patient sex	36.1% (*n* = 169) male: 63.9% (*n* = 299) female
Patient age (mean, *SD*)	79.1 (*SD* = 8.1)
FI concentration (range, mean, *SD*)	6.2–36 μg/ml, 19.6 μg/ml (*SD* = 5.1)
CRP concentration (range, mean, *SD*)	0.3–59 mg/L, 4.2 mg/L (*SD* = 6.5)
Patients with low serum FI level less than 15.6 μg/ml	19.4% of total recruited patients (*n* = 91)
Patients with low serum FI level and rare *CFI* gene variants detected^a^	4.7% of total recruited patients (*n* = 22)
Patients with pathogenic or likely pathogenic *CFI* gene variants^a^	3.2% of total recruited patients (*n* = 15)
Prevalence of rare *CFI* gene variants in patients with low serum FI level and GA	25%

Abbreviations: CFI, complement factor I; FI, factor I; GA, geographic atrophy.

^a^ All patients in whom a low serum FI level and rare *CFI* gene variant were both detected, described their ethnicity as “White: British”.

Altogether, 19.4% (*n* = 91) of total recruited patients had a serum FI concentration below 15.6 μg/ml, designated as the lower limit of normal plasma FI concentration in this study (see Section 2). *CFI* gene sequencing was undertaken in all patients with a low serum FI concentration (*n* = 88, 3 patients having withdrawn from the study before sequencing). Rare *CFI* gene variants, with a MAF of less than 1%, were detected in 22 patients (4.7% of total recruited patients). The prevalence of rare *CFI* gene variants in patients with low serum FI levels was thus 25%. All patients in whom a low serum FI level and rare *CFI* gene variant were both detected, described their ethnicity as “White: British”. There was no statistically significant difference in age between those patients with a *CFI* gene variant (average age = 78.18; *SD* = 6.18; *n* = 22) and those without a *CFI* gene variant (average age = 79.11; *SD* = 8.16; *n* = 446) using an unpaired, two‐tailed, *t* test (*p* = .5072). The percentage of patients with a rare *CFI* gene variant who were male was 27.3% (*n* = 6). The percentage of females was 72.7% (*n* = 16). Using a 2 × 2 contingency table, and a Fisher's exact test, there was no statistically significant association detected between the expression of a rare *CFI* gene variant and the sex of the patient (*p* = .4968).

The serum FI concentrations of 88 patients with low serum FI levels (<15.6 μg/ml) were displayed graphically according to the detection of rare *CFI* gene variants (Figure 2b). There was a statistically significant difference in the mean serum FI concentration of those patients with a *CFI* gene variant (mean = 10.2 μg/ml; *SD* = 2.6; *n* = 22) and those without a *CFI* variant (mean = 14.2 μg/ml; *SD* = 1.2; *n* = 66) using an unpaired *t* test with Welch's correction (*p *< .001). Patients with a rare *CFI* gene variant demonstrated an additional reduction in serum FI levels compared to those patients without a *CFI* gene variant.

The serum CRP concentration of patients recruited to this study with a low serum FI level and GA was also investigated. There was a statistically significant difference detected in mean serum CRP concentration between patients with a low serum FI concentration and a *CFI* gene variant (mean = 2.3 mg/L; *SD* = 1.5; *n* = 13) and those expressing normal serum FI levels ≥15.6 μg/ml (mean = 4.7 mg/L; *SD* = 7.0; *n* = 266), based on an unpaired, two‐tailed *t* test with Welch Correction (*p* = .003) (Figure 2c). Only those patients with a clinically detectable CRP more than 0.3 mg/L were used in statistical analysis. Furthermore, there was also a statistically significant difference detected in mean serum CRP concentration between patients with a low serum FI level without a *CFI* gene variant (mean = 1.747 mg/L; *SD* = 1.815; *n* = 43) and patients expressing serum FI level ≥15.6 μg/ml (*p* < .0001).

### 3.2 Genotype analysis: bioinformatic prediction of rare *CFI* gene variant pathogenicity

Twenty‐one patients in this study with a low serum FI concentration, in whom a rare *CFI* gene variant was detected, were invited for a second visit to undergo a fundus examination and retinal imaging (one patient died before visit two). All 21 patients with *CFI* gene variants are listed in (Table 2) together with the corresponding nucleotide and protein change, in addition to MAF. Classification of the pathogenicity of the *CFI* variant, based on bioinformatic prediction and American College of Medical Genetics and Genomics (ACMG) guidelines, is listed with associated references. Of these 21 patients, 15 expressed a rare *CFI* gene variant that is pathogenic or likely pathogenic. This equates to 3.2% of total patients recruited to this study. The largest proportion of these patients (*n* = 10) expressed the *CFI* p.Gly119Arg (NM_000204.3:c.355G>A) variant. Four patients expressed the following rare pathogenic *CFI* gene variants: p.Pro50Ala (NM_000204.3:c.148C>G), p.Ala258Thr (NM_000204.3:c.772G>A), p.His418Leu (NM_000204.3:c.1253A>T), and p.Ala431Thr (NM_000204.3:c.1291G>A). Additionally, a fifth patient expressed a rare *CFI* gene variant, which is predicted to be pathogenic: p.Arg502Cys (NM_000204.3:c.1504C>T).

**Table 2:** Genotype‐phenotype analysis of 21 patients with geographic atrophy and low serum factor I concentration (<15.6 μg/ml) in whom a rare *CFI* gene variant was detected on DNA sequencing

Patient ID	Patient age	*CFI* gene variant (HGVS)	Protein change	Serum FI (μg/ml)	Reticular pseudodrusen	Bioinformatic /ACMG Classification^a^	Minor allele frequency^b^
SUN035	79	NM_000204.3:c.148C>G	p.Pro50Ala	14.7	Yes	Pathogenic variant	0.0001008
BEL037	75	NM_000204.3:c.355G>A	p.Gly119Arg	9.3	Yes	Pathogenic variant	0.0008519
BRI024	84	NM_000204.3:c.355G>A	p.Gly119Arg	8.1	Yes	Pathogenic variant	0.0008519
BRI044	62	NM_000204.3:c.355G>A	p.Gly119Arg	9.4	Yes	Pathogenic variant	0.0008519
GLO023	79	NM_000204.3:c.355G>A	p.Gly119Arg	9.8	Yes	Pathogenic variant	0.0008519
MAN001	81	NM_000204.3:c.355G>A	p.Gly119Arg	7.7	Yes	Pathogenic variant	0.0008519
MAN033	82	NM_000204.3:c.355G>A	p.Gly119Arg	10.2	Yes	Pathogenic variant	0.0008519
MOR036	75	NM_000204.3:c.355G>A	p.Gly119Arg	9.4	Yes	Pathogenic variant	0.0008519
OXF002	79	NM_000204.3:c.355G>A	p.Gly119Arg	9.5	Yes	Pathogenic variant	0.0008519
SOU001	84	NM_000204.3:c.355G>A	p.Gly119Arg	8	Yes	Pathogenic variant	0.0008519
SUN013	71	NM_000204.3:c.355G>A	p.Gly119Arg	10.9	Yes	Pathogenic variant	0.0008519
SUN008	73	NM_000204.3:c.772G>A	p.Ala258Thr	11.2	Yes	Pathogenic variant	0.0002400
MOR010	81	NM_000204.3:c.1253A>T	p.His418Leu	7.2	Yes	Pathogenic variant	0.00002638
SUN038	87	NM_000204.3:c.1291G>A	p.Ala431Thr	7.1	Yes	Pathogenic variant	0.00004398
BRI050	66	NM_000204.3:c.1504C>T	p.Arg502Cys	12.2	Yes	Likely Pathogenic	0.000007748
OXF005	84	NM_000204.3:c.912A>G	p.Thr304=	14.1	Yes	Uncertain clinical significance	0.00004401
BRI012	85	NM_000204.3:c.782G>A	p.Gly261Asp	9.4	Yes	Uncertain clinical significance	0.001920
BRI039	77	NM_000204.3:c.1216C>T	p.Arg406Cys	14.4	No	Uncertain clinical significance	0.00002324
MAN037	80	NM_000204.3:c.782G>A	p.Gly261Asp	6.2	Yes	Uncertain clinical significance	0.001920
BEL017	76	NM_000204.3:c.1548T>C	p.Gly516=	13.3	Yes	Uncertain clinical significance	0.0001552
MAN004	83	NM_000204.3:c.782G>A	p.Gly261Asp	8	No	Uncertain clinical significance	0.001920

Abbreviations: ACMG, American College of Medical Genetics and Genomics; CFI, complement factor I; FI, factor I; HGVS, human genome variation society nomenclature.

^a^ See Section 4 for associated references for Bioinformatic/ACMG Classification of *CFI* gene variants.

^b^ Minor allele frequency based on population frequency (European, non‐Finnish) according to the genome aggregation database (gnomAD).

A total of 6 of 21 patients with low serum FI concentrations expressed a rare *CFI* gene variant of uncertain clinical significance: Three patients expressed the *CFI* p.Gly261Asp variant (NM_000204.3:c.782G>A), one patient expressed the p.Arg406Cys variant of *CFI* (NM_000204.3:c.1216C>T), and two patients expressed synonymous *CFI* gene variants: p.Thr304= (NM_000204.3:c.912A>G) and p.Gly516= (NM_000204.3:c.1548T>C).

### 3.3 Phenotype analysis: common detection of RPD in patients with pathogenic rare *CFI* gene variants

**Figure 3:**
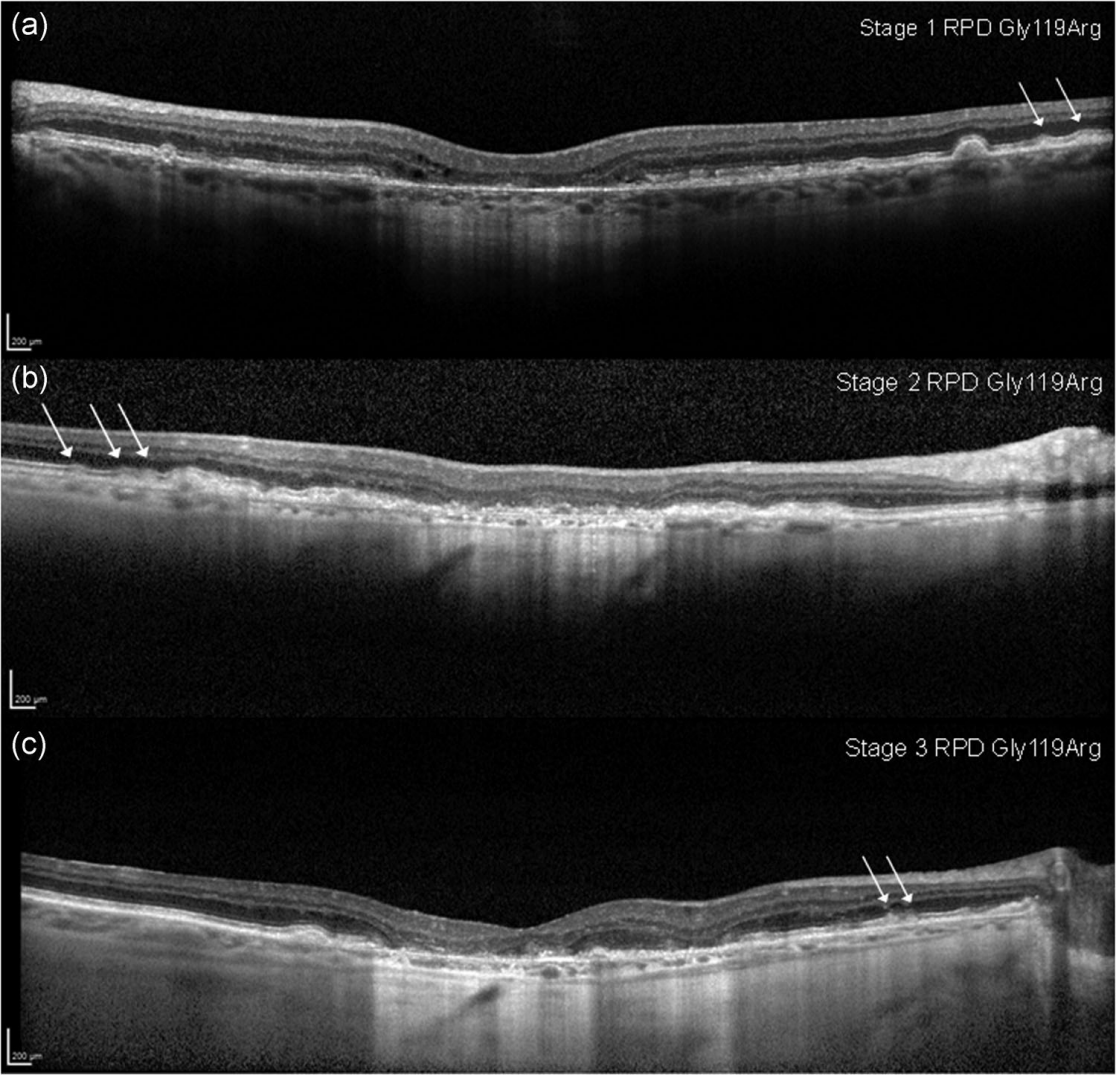
Representative examples of SD‐OCT images from the right eye of patients with low serum FI levels, demonstrating both geographic atrophy and different stages of reticular pseudodrusen (white arrows) according to the Querques et al. (2012; 2013) classification. All patients from whom the above SD‐OCT images were taken expressed the p.Gly119Arg (NM_000204.3:c.355G>A) *CFI* gene variant. The display of reticular pseudodrusen includes Stage 1 RPD: deposition of hyper‐reflective material between the RPE and ellipsoid zone (EZ) (a); Stage 2 RPD: Mounds of accumulated hyper‐reflective material to alter the contour of the EZ (b); Stage 3 RPD**:** Thicker material that adopts a more conical shape and breaks through the EZ (c)

Retinal images taken of the 21 GA patients with low serum FI levels and expression of a rare *CFI* gene variant were reviewed by up to three ophthalmologists and a consensus was reached on phenotypic properties. A common property was the presence of RPD in all 15 patients with GA expressing a pathogenic or likely pathogenic rare *CFI* gene variant described above and listed in Table 2. This included all patients with the p.Gly119Arg *CFI* variant (NM_000204.3:c.355G>A). Four of the six patients expressing a *CFI* gene variant of uncertain clinical significance (based on bioinformatic prediction), also expressed RPD. Representative SD‐OCT images of patients with low serum FI, that display both GA and stages of RPD (Querques et al. [2012; 2013] classification) on the same OCT frame are shown (Figure 3). All three patients in the images shown in Figure 3 have the pathogenic p.Gly119Arg *CFI* gene variant. Despite all 15 patients with a pathogenic or likely pathogenic *CFI* gene variant expressing RPD, a statistically significant association could not be reached between these variants (15 of 21 patients with a *CFI* gene variant) and RPD using a 2 × 2 contingency table and a Fisher's exact test with a two‐sided *p* value (*p *= .0714). The comparison group was the remaining group of six patients with *CFI* gene variants of uncertain clinical significance.

### 3.4 Phenotype analysis: effect of rare *CFI* gene variant pathogenicity on serum FI concentration

The serum FI concentrations of 88 patients with GA and low serum FI levels (<15.6 μg/ml) were displayed graphically according to rare *CFI* gene variant pathogenicity. There was a statistically significant difference in mean serum FI concentration between patients expressing a pathogenic *CFI* gene variant (mean = 9.6 μg/ml; *SD* = 2.0; *n* = 15) and patients expressing no *CFI* variants (mean = 14.2 μg/ml; *SD* = 1.2; *n* = 66), based on an unpaired, two‐tailed *t* test with Welch Correction (*p* < .0001) (Figure 2d). Patients with a *CFI* variant demonstrated an additional reduction in mean serum FI level. The mean serum FI concentration of all 468 patients recruited to the study was 19.6 μg/ml (*SD* = 5.1). The difference in mean serum FI concentration between patients expressing a rare *CFI* gene variant of unknown clinical significance (mean = 10.9 μg/ml; *SD* = 3.5; *n* = 6) and those expressing no *CFI* gene variants did not reach statistical significance (*p* = .0691).

### 3.5 *CFI* gene variants and association with SNPs at 10 common AMD loci and Haplotypes

All patients recruited to this study with GA and a serum FI concentration less than 15.6 μg/ml (*n* = 88) underwent DNA sequencing at 10 common AMD loci in addition to the sequencing of the *CFI* gene. Sequencing results were used to investigate: (1) an association between rare *CFI* gene variants and SNPs at the *CFI* region of chromosome 4, which could suggest linkage disequilibrium; and (2) an association between rare *CFI* gene variants and complement risk SNPs at common AMD loci that could suggest the influence of overall complotype on FI activity and the alternative complement pathway in GA (Lay et al., 2015). The following 10 susceptibility SNPs associated with AMD were sequenced (human genome variation society nomenclature stated in Section 2): *CFH* region: rs800292; rs1061170; rs10737680; rs1329428 (Fritsche et al., 2016; Raychaudhuri et al., 2011); *CFI* region: rs17440077; rs4698775; rs2285714 (Chen et al., 2010; Fagerness et al., 2009; Fritsche et al., 2016); *CFB* region: rs429608 (Shuai et al., 2017); *ARMS2* region: rs10490924 (Rivera et al., 2005); and *C3* region: rs2230199 (Maller et al., 2007). Each SNP and its associated chromosome number, including notable or nearby DNA region is listed in (Table S1). Single‐variant association studies were performed using 2 × 2 contingency tables, Fischer's exact test, and two‐tailed *p* values to investigate associations between *CFI* gene variants and complement risk SNPs at the 10 AMD loci for the reasons stated above. The comparison group was patients without the stated risk SNP at each locus. The only significant *p* value (.0113) achieved was for the presence of SNP rs2285714 at the *CFI* region (*PLA2G12A*: c.345G>A), but this was in the absence of a rare *CFI* gene variant (Table S1): there were 52 cases of this SNP in the absence of a *CFI* gene variant compared to 10 cases of this SNP in the presence of a *CFI* gene variant. However, this did not reach statistical significance after a Bonferroni correction was applied (*p* value was set at <.005). No statistically significant associations were otherwise reached.

Contingency tables were also used to extend the question above: to investigate an association between *CFI* gene variants and homozygosity for risk SNPs at the 10 common AMD loci sequenced in this study that could suggest the influence of overall complotype on FI activity and the alternative complement pathway in GA (Table S2). The comparison group was those patients without SNP homozygosity. A *p* value of 0.0341 was achieved with homozygosity of SNP rs2285714 at the *CFI* region (*PLA2G12A*: c.345G>A), but this was in the absence of a rare *CFI* gene variant: there were 18 cases of homozygosity of this SNP in the absence of a *CFI* gene variant compared to one case of SNP homozygosity in the presence of a *CFI* gene variant. After Bonferroni correction, this did not reach statistical significance (*p* value was set at <.006). No other statistically significant associations were reached. Last, contingency tables were used to investigate associations between *CFI* gene variant expression and four different haplotypes, which each include SNPs at two common AMD loci (Table S3). No statistically significant associations were reached.

## 4 DISCUSSION

The CFI study recruited 468 patients from 12 UK hospitals. The aim of the study was to identify the prevalence of patients with GA who express low systemic levels of FI, in addition to those with rare *CFI* gene variants. The study also aimed to perform a genotype‐phenotype study for these patients to identify patients who could be eligible for future therapeutic trials. The significant findings of the study were as follows: 19.4% of recruited patients (*n* = 91) demonstrated a low serum FI concentration below 15.6 μg/ml (definition of threshold described in the methods section). *CFI* gene sequencing undertaken on all patients with a low serum FI concentration detected rare *CFI* gene variants in 4.7% of total recruited patients with GA. The prevalence of *CFI* variants in patients with low serum FI levels and GA was 25%. There was no association detected between either age or sex and the presence of *CFI* gene variants in these patients. Patients with low serum FI levels and a rare *CFI* gene variant demonstrated an additional reduction in serum FI concentration that was statistically significant compared to those patients without a *CFI* gene variant.

Furthermore, 3.2% of total patients recruited to this study (*n* = 15) expressed a pathogenic or likely pathogenic *CFI* gene variant. The largest proportion of these patients (*n* = 10) expressed the *CFI* p.Gly119Arg variant (NM_000204.3:c.355G>A), which has robust evidence of pathogenicity in previous studies (de Jong et al., 2020; Fremeaux‐Bacchi et al., 2013; Hallam et al., 2020; Kavanagh et al., 2015; Maga et al., 2010; van de Ven et al., 2013). Four patients expressed a rare pathogenic *CFI* gene variant, which also resulted in low serum FI levels in previous studies: p.Pro50Ala (Bienaime et al., 2010; Nilsson et al., 2010; Szilagyi et al., 2013), p.Ala258Thr (Alba‐Dominguez et al., 2012; Kavanagh et al., 2015; Nilsson et al., 2009; Ponce‐Castro et al., 2008; Sullivan et al., 2010; Vyse et al., 1996), p.His418Leu (Donegan et al., 2020; Nilsson et al., 2009; Vyse et al., 1996), and p.Ala431Thr (Bienaime et al., 2010). A fifth patient expressed the p.Arg502Cys *CFI* gene variant (Kavanagh et al., 2015; Seddon et al., 2013) that was classified as likely pathogenic based on bioinformatics and ACMG guidelines. Six patients with low FI levels in this study expressed rare *CFI* gene variants of uncertain clinical significance based on previous published reports. Three of these patients with low FI levels (6.2, 8, and 9.4 μg/ml) expressed the *CFI* p.Gly261Asp variant. Interestingly, this is in contrast to previous studies in which this variant was associated with normal serum FI levels (Hallam et al., 2020; Kavanagh et al., 2008; Kavanagh et al., 2015; Nilsson et al., 2007) (although splicing changes have been predicted; Xiong et al., 2015). It is likely that these low FI readings represent false‐positive findings as this contrasts with multiple previously published reports, and we hypothesize that the variant interferes with the epitope of the antibody used in this ELISA. Additionally, one patient expressed the p.Arg406Cys variant of *CFI* (Geerlings et al., 2017; Roversi et al., 2011), and two others expressed the p.Gly516= and p.Thr304= variants (synonymous changes). None of these variants have previously been reported to result in low serum FI concentrations, contrasting with this study. As this study used low serum FI concentrations as the starting point to select patients with GA for *CFI* gene sequencing, it is entirely possible that patients with rare *CFI* gene variants, which result in normal FI levels but dysfunctional FI protein were not detected. Therefore, a limitation of this study was restricting DNA sequencing to patients with a reduced serum FI concentration. If all patients underwent DNA sequencing, to detect those *CFI* gene variants that result in normal FI levels but dysfunctional protein, functional analyses would have been required to detect the biological effect of these gene variants on the alternative complement pathway, and this was beyond the scope of this study.

This study also assessed whether there was an association between rare *CFI* gene variants and risk SNPs at 10 common AMD loci (including complement pathway SNPs). This could suggest influence of overall complotype on FI levels or activity in all patients with GA and a serum FI concentration less than 15.6 μg/ml (*n* = 88). After Bonferroni correction, no statistically significant associations were observed in these patients using single‐variant studies. Furthermore, as the risk SNPs for *CFH* (rs1061170) and *ARMS2* (rs10490924) have a disproportionately larger AMD risk relative to other common SNPs tested, we reviewed their distribution amongst patients with *CFI* gene variants. Interestingly, of 22 patients with rare *CFI* gene variants, the *CFH* SNP was detected in 73% (*n* = 16) of them, with 27% (*n* = 6) being homozygous. In addition, the *ARMS2* SNP was detected in 68% (*n* = 15) of these patients, with 9% (*n* = 2) being homozygous. However, there was no statistically significant association observed when assessing all GA patients with reduced serum FI concentration.

In this study, of the 91 patients with GA that had low serum FI concentrations, a rare *CFI* gene variant was detected in only 22 of them. It is possible that in patients without rare *CFI* gene variants, low serum FI concentrations were the result of nongenetic factors, such as consumption of FI due to increased complement activity. Increased complement activity is well known in advanced AMD patients (Reynolds et al., 2009; Scholl et al., 2008). It has also been noted in systemic lupus erythematosus (SLE) that FI serum levels are lower during SLE active phases compared to recessive phases in patients, suggesting complement‐mediated FI consumption during disease (Tseng et al., 2018). Furthermore, the assay used in our study may not have been sufficiently sensitive to find all genetic variants, for example, intronic sequences/noncoding DNA. It is also possible that systemic levels of FI were influenced by *CFI* gene region SNPs that were tested for as part of the 10 common AMD SNPs. A large number of patients expressed the *CFI* region SNPs rs17440077, rs4698775, or rs2285714, which did not express rare *CFI* gene variants. The statistical tests undertaken in the study were only to detect associations between rare *CFI* gene variants and each of the common AMD SNPs. Last, another possible reason for not finding gene variants in patients with low FI levels was that the lower limit of normal FI concentration established by our preliminary AMD cohort (see Section 2) was too high due to the 18% assay variation in addition to the lower 2.5th percentile of serum FI concentrations. Further clinical studies into *CFI* variants and FI supplementation will further refine this threshold.

Interestingly, a phenotypic feature common to every patient in this study with low serum FI levels and a pathogenic *CFI* gene variant, was the presence of RPD on retinal imaging. This included every patient (*n* = 10) expressing the p.Gly119Arg *CFI* variant. 90.5% (*n* = 19) of all GA patients in this study expressing low serum FI levels and any *CFI* gene variant displayed RPD on imaging. A statistically significant association could not be reached between pathogenic or likely pathogenic *CFI* gene variants and the presence of RPD, compared to those patients with rare *CFI* gene variants that were not classified as pathogenic (a small group of six patients). With advances in retinal imaging, clinical studies have revealed the presence of RPD to be an independent risk factor in AMD progression (Marsiglia et al., 2013; Sivaprasad et al., 2016). In contrast to the hallmark drusenoid deposits (drusen) seen in AMD, which are located between the RPE and the inner collagenous layer of Bruch's membrane, RPD are subretinal drusenoid deposits that are located internal to the RPE (Curcio et al., 2013; Spaide & Curcio, 2010). The presence of RPD has been shown to confer a 4–8 fold greater risk of 5‐year progression to the advanced stages of AMD, including GA, than the presence of drusen alone (Joachim et al., 2014). Furthermore, RPD has been demonstrated to coexist with the presence of GA or nAMD (Marsiglia et al., 2013; Schmitz‐Valckenberg et al., 2011; Zweifel et al., 2010). The presence of RPD has been associated with the *CFI* p.Gly119Arg substitution in a previous study, although this was shown in families with AMD rather than a large AMD Cohort (Saksens et al., 2016). Furthermore, RPD was noted in both eyes of approximately 15% of AMD patients in a post hoc analysis of the CATT Trial (Lin et al., 2018). It has been speculated that RPD may be a manifestation of the failure to regulate age‐associated RPE damage via para‐inflammation, and furthermore, might be a feature of heightened immune responses driving RPE and retinal damage (Sivaprasad et al., 2016). In addition to RPD being a well reported, coexisting feature in GA (described above), another possible explanation for the common detection of RPD in patients with pathogenic *CFI* gene variants is that they have advanced Bruch's membrane damage. RPD are present consistently in diseases with primary pathology in Bruch's membrane, such as pseudoxanthoma elasticum (Gliem et al., 2015a), Sorsby fundus dystrophy (Gliem et al., 2015b), and late‐onset retinal degeneration (Cukras et al., 2016). 

Both CRP and FI are acute phase reactants. CRP is a marker of systemic inflammation that is an independent risk factor for AMD (Seddon et al., 2004). Serum CRP levels were measured in all patients recruited to this study to ensure that FI concentrations were standardized and not raised artefactually due to systemic inflammation (which would also have resulted in raised CRP levels). In line with the function of CRP and FI as acute phase reactants, patients in this study with low serum CRP levels demonstrated lower serum FI levels. Furthermore, there was a positive correlation detected between serum concentrations of CRP and FI in patients with GA. As FI functions as an inhibitor of complement activation, one would expect raised systemic FI levels during an acute inflammatory event. It is interesting to note that although CRP is an independent risk factor for AMD, it correlates positively with levels of protective FI in this study.

The assessment of rare *CFI* gene variant frequency associated with low FI levels in patients with GA could help stratify patients who are at the early, presymptomatic stages of AMD, but demonstrate risk factors for disease progression. These patients could be eligible for future therapeutic trials. Highly penetrant, rare complement pathway SNPs, including the p.Gly119Arg *CFI* variant (with associated strong effect size), have been identified in families with AMD, in line with the hypothesis that rare variants cluster in families (Yu et al., 2014). This raises the question of a founder effect. However, we have no data, such as linkage disequilibrium data, to support or refute this suggestion. More recent studies have demonstrated that this variant contributes to an earlier age of AMD onset and progression than in noncarriers (Saksens et al., 2016). Accordingly, any participant in this study with an identified rare *CFI* variant during sequencing was informed of this result (as per the consent process), so that they could inform family members to undergo an ophthalmic examination under the National Health Service.

This study raises the prospect that patients with early features of AMD (including those with high‐risk features such as RPD) could undergo a blood test to measure serum FI levels, and those expressing low serum FI levels and pathogenic rare *CFI* gene variants could potentially benefit from an early FI supplementation approach. This would most likely be a cost‐effective and time‐saving method than undertaking DNA sequencing on all patients. *CFI* gene sequencing would cost approximately 300 GBP (400 USD) per patient, involving DNA extraction, PCR, purification, sequencing, and multiplex ligation‐dependent probe amplification, followed by analysis. This could take up to approximately 2 weeks if several patient samples have to be analyzed. In contrast, an ELISA for FI takes approximately 1 day to perform and would cost approximately 40 GBP (50 USD). The fact that this study was able to recruit 486 patients on a limited budget attests to the practicality of this approach.

According to a meta‐analysis of AMD prevalence applied to 2007–2009 UK data, the prevalence of late AMD in the UK was 513,000 cases, of which 276,000 cases were due to GA (Owen et al., 2012). As the p.Gly119Arg was the most common pathogenic *CFI* variant detected in this study (known to result in reduced FI levels), and has a population frequency of 0.0008519 (European, non‐Finnish) according to the genome aggregation database (gnomAD), it is estimated that approximately 235 cases of GA (0.08%) could be attributed to this gene variant in the United Kingdom. The annual number of incident cases of GA in the UK has been estimated at 43,700 (Owen et al., 2012). In light of the significant morbidity associated with late stage AMD, it would be cost‐effective to measure FI levels in asymptomatic AMD patients (including those with high risk features), and assess whether low FI is a result of pathogenic *CFI* variants, and amenable to FI supplementation to reduce the risk of progression to GA. As a recent Australian cohort study has shown that early AMD is not always significantly progressive because 83% did not progress (Keel et al., 2017), it would be economically challenging to undertake whole genome sequencing or even single‐gene analysis on such a large volume of patients as detailed above. However, if funding was available, such a DNA sequencing approach may detect more patients with rare *CFI* variants cf. the approach adopted in this study. Therefore, depending on resources this could be considered too.

In summary, this study demonstrates for the first time, the prevalence of disease‐causing *CFI* gene mutations in an unselected cohort of GA patients. It highlights an association with RPD in these patients. It demonstrates the feasibility of a two‐stage approach to identify those patients who would be most amenable to FI supplementation as a novel therapeutic approach to treat GA.

## CONFLICT OF INTERESTS

**Adnan H. Khan**: none; **Janice Sutton**: None; **Angela Cree**: none; **Samir Khandhadia**: none; **Gabriella De Salvo**: none; **John Tobin**: Gyroscope Therapeutics (Employee); **Rashi Arora**: none; **Geeta Menon**: none; **Priya Prakash**: none; **Tunde Peto**: none; **Quresh Mohamed**: Allergan (Consult), Bayer (Consult), Novartis (Consult), Roche (Consult); **Winfried Amoaku**: Bayer Int (Grant), Novartis (Interest), Boerhinger Ingelheim (Grant), Abbvie/Allergan (Interest), Roche (Interest); **Clare Bailey**: Bayer (Consult), Novartis (Consult), Roche (Consult), Alimera Sciences (Consult), **David Steel**: Gyroscope Therapeutics (Interest; Consult), Alcon (Grant; Interest), Bayer (Grant), Roche (Interest); **Sobha Sivaprasad**: Allergan (Grant; Interest), Bayer (Grant; Interest), Novartis (Grant; Interest), Boerhinger Ingelheim (Grant; Interest), Oxurion (Interest), Apellis (Interest), Heidelberg Engineering (Interest), Optos (Grant; Interest); **Paul N. Bishop**: Complement Therapeutics (Interest; Director; Founder); **Peter Charbel Issa**: Gyroscope Therapeutics (Consult); **Robert E. MacLaren**: Gyroscope Therapeutics (Interest; Consult); **David Kavanagh**: Gyroscope Therapeutics (Interest; Consult); **Andrew J. Lotery**: Gyroscope Therapeutics (Interest; Consult).

## WEB RESOURCES

Genome aggregation database (gnomAD): http://gnomad.broadinstitute.org.

## Supporting information

Supplementary information.

## Data Availability

The data that support the findings of this study are available from the corresponding author upon reasonable request.
