# Supplementary material for: Prevalence and phenotype associations of complement factor I mutations in geographic atrophy
Source: Hum Mutat. 2021 Jun 29;42(9):1139–52. doi: 10.1002/humu.24242 (PMC9290714; doi:10.1002/humu.24242)
Supplement: Supplementary file 1 — Supplementary information. [file HUMU-42-1139-s001.pdf]

Supplementary Table 1. *CFI* gene variants in patients with Geographic Atrophy and low serum FI concentration (<15.6 µg/ml), and association with SNPs at common AMD loci

**Notable or Nearby DNA region**	**Reference SNP**	**Gene Variant (HGVS)**	**No *CFI* gene variant, *n***	***CFI* gene variant, *n***	***P* value**
Chr1: *CFH*	rs800292	NM_000186.4:c.184G>A	13	2	0.507
Chr1: *CFH*	rs1061170	NM_000186.4:c.1204C>A	40	16	0.2954
Chr1: *CFH*	rs10737680	NC_000001.11:g.196710325A>C	21	6	1
Chr1: *CFH*	rs1329428	NC_000001.11:g.196733680C>T	21	6	1
Chr4: *CFI* region (*MCUB*)	rs17440077	NC_000004.12:g.109616411A>G	46	11	0.189
Chr4: *CFI* region (*MCUB*)	rs4698775	NC_000004.12:g.109669323G>T	53	19	0.507
Chr4: *CFI* region (*PLA2G12A*)	rs2285714	NM_030821.5:c.345G>A	52	10	**0.0113**
Chr6: *CFB* region (*SKIV2L*)	rs429608	NC_000006.12:g.31962685G>A	10	3	1
Chr10: *ARMS2*	rs10490924	NM_001099667.3:c.205G>T	38	15	0.3113
Chr19: *C3*	rs2230199	NM_000064.4:c.304C>G	32	10	1

SNP = Single Nucleotide Polymorphism; HGVS = Human Genome Variation Society Nomenclature; CFI = Complement Factor I. Single-variant association studies were performed using 2 x 2 contingency tables, Fischer's exact test and two-tailed *P* values. Statistical significance was defined as $P < 0.05$.

**Supplementary Table 2.** ***CFI*** **gene variants in patients with Geographic Atrophy and low serum FI concentration (<15.6µg/ml), and association with Homozygosity for SNPs at common AMD loci**					
**Notable or Nearby DNA region**	**Reference SNP**	**Gene Variant (HGVS)**	**No** ***CFI*** **gene variant,** ***n***	***CFI*** **gene variant,** ***n***	***P*** **value**
Chr1: *CFH*	rs800292	NM_000186.4:c.184G>A	1	0	1
Chr1: *CFH*	rs1061170	NM_000186.4:c.1204C>A	12	6	0.3574
Chr1: *CFH*	rs10737680	NC_000001.11:g.196710325A>C	5	0	0.3303
Chr1: *CFH*	rs1329428	NC_000001.11:g.196733680C>T	5	0	0.3303
Chr4: *CFI* region (*MCUB*)	rs17440077	NC_000004.12:g.109616411A>G	8	2	1
Chr4: *CFI* region (*MCUB*)	rs4698775	NC_000004.12:g.109669323G>T	23	10	0.313
Chr4: *CFI* region (*PLA2G12A*)	rs2285714	NM_030821.5:c.345G>A	18	1	**0.0341**
Chr10: *ARMS2*	rs10490924	NM_001099667.3:c.205G>T	16	2	0.2185
Chr19: *C3*	rs2230199	NM_000064.4:c.304C>G	5	1	1

SNP = Single Nucleotide Polymorphism; HGVS = Human Genome Variation Society Nomenclature; CFI = Complement Factor I. Single-variant association studies were performed using 2 x 2 contingency tables, Fischer's exact test and two-tailed *P* values. Statistical significance was defined as $P < 0.05$.

Supplementary Table 3. *CFI* gene variants in patients with Geographic Atrophy and low serum FI concentration (<15.6µg/ml), and association with Haplotypes of common AMD SNPs

**Reference SNP combinations<br>(Notable or Nearby Gene Region), HGVS**	**No *CFI* gene variant, *n***	***CFI* gene variant, *n***	***P* value**
rs1061170 (*CFH*),<br>NM_000186.4:c.1204C>A<br>and<br>rs4698775 (*CFI* region*: MCUB*),<br>NC_000004.12:g.109669323G>T	33	15	0.13
rs1061170 (*CFH*),<br>NM_000186.4:c.1204C>A<br>and<br>rs17440077 (*CFI* region: *MCUB*),<br>NC_000004.12:g.109616411A>G	29	9	1
rs1061170 (*CFH*),<br>NM_000186.4:c.1204C>A<br>and<br>rs2285714 (*CFI* region: *PLA2G12A*),<br>NM_030821.5:c.345G>A	34	9	0.6177
rs17440077 (*CFI* region: *MCUB*),<br>(NC_000004.12:g.109616411A>G)<br>and<br>rs4698775 (*CFI* region: *MCUB*),<br>(NC_000004.12:g.109669323G>T)	39	9	0.2165

SNP = Single Nucleotide Polymorphism; HGVS = Human Genome Variation Society Nomenclature; CFI = Complement Factor I. Single-variant association studies were performed using 2 x 2 contingency tables, Fischer's exact test and two-tailed *P* values. Statistical significance was defined as $P < 0.05$.
